# Diversity and plant growth-promoting potential of (un)culturable bacteria in the *Hedera helix* phylloplane

**DOI:** 10.1186/s12866-021-02119-z

**Published:** 2021-02-27

**Authors:** Vincent Stevens, Sofie Thijs, Jaco Vangronsveld

**Affiliations:** 1 Center for Environmental Sciences, Environmental Biology, Hasselt University, Diepenbeek, Belgium; 2 Department of Plant Physiology, Faculty of Biology and Biotechnology, Maria Curie–Skłodowska University, Lublin, Poland

**Keywords:** *Hedera helix*, Phylloplane, Microbial diversity, Culture-independent, Culturing, Growth media, Plant growth promotion

## Abstract

**Background:**

A diverse community of microbes naturally exists on the phylloplane, the surface of leaves. It is one of the most prevalent microbial habitats on earth and bacteria are the most abundant members, living in communities that are highly dynamic. Today, one of the key challenges for microbiologists is to develop strategies to culture the vast diversity of microorganisms that have been detected in metagenomic surveys.

**Results:**

We isolated bacteria from the phylloplane of *Hedera helix* (common ivy), a widespread evergreen, using five growth media: Luria–Bertani (LB), LB01, yeast extract–mannitol (YMA), yeast extract–flour (YFlour), and YEx. We also included a comparison with the uncultured phylloplane, which we showed to be dominated by Proteobacteria, Actinobacteria, Bacteroidetes, and Firmicutes. Inter-sample (beta) diversity shifted from LB and LB01 containing the highest amount of resources to YEx, YMA, and YFlour which are more selective. All growth media equally favoured Actinobacteria and Gammaproteobacteria, whereas Bacteroidetes could only be found on LB01, YEx, and YMA. LB and LB01 favoured Firmicutes and YFlour was most selective for Betaproteobacteria. At the genus level, LB favoured the growth of *Bacillus* and *Stenotrophomonas*, while YFlour was most selective for *Burkholderia* and *Curtobacterium*. The in vitro plant growth promotion (PGP) profile of 200 isolates obtained in this study indicates that previously uncultured bacteria from the phylloplane may have potential applications in phytoremediation and other plant-based biotechnologies.

**Conclusions:**

This study gives first insights into the total bacterial community of the *H. helix* phylloplane, including an evaluation of its culturability using five different growth media. We further provide a collection of 200 bacterial isolates underrepresented in current databases, including the characterization of PGP profiles. Here we highlight the potential of simple strategies to obtain higher microbial diversity from environmental samples and the use of high-throughput sequencing to guide isolate selection from a variety of growth media.

**Supplementary Information:**

The online version contains supplementary material available at 10.1186/s12866-021-02119-z.

## Background

An abundant and diverse community of microorganisms naturally exists on the surface of above-ground parts of plants, known as the phyllosphere [1]. The phyllosphere can be subdivided into the caulosphere (stems), phylloplane (leaves), anthosphere (flowers), and carposphere (fruits). The phyllosphere is one of the most prevalent microbial habitats on earth and bacteria are by far the most abundant and persistent phyllosphere organisms, with a typical cell density of 10^6^–10^7^ cells cm^− 2^ [1, 2]. Phyllosphere microbial community studies to date have mainly focused on plant species such as *Arabidopsis thaliana* (thale cress), *Lactuca sativa* (lettuce), *Glycine max* (soy bean), *Trifolium repens* (white clover), and *Oryza sativa* (rice) and the greatest microbial diversity has been described using metagenomic tools. Broadly, leaf microbial communities mainly comprise bacteria belonging to the phyla Proteobacteria, Actinobacteria, Bacteroidetes, and Firmicutes. Further, Proteobacteria species have been reported to comprise about half of the phyllosphere community suggesting that, at higher taxonomic levels, phyllosphere bacterial communities are similar across various host plant species [1, 3–8]. Increased knowledge of plant–microbe interactions enables a better understanding of their role during natural plant growth and development [9], and this knowledge can be translated into improved agricultural biomass production and microbe-assisted phytotechnologies [10]. In this study, the bacterial phylloplane community of *Hedera helix* (common ivy) is explored using culture-dependent and -independent techniques. *H. helix* is an evergreen plant known for its hardiness and climbing ability [11], and has widespread distribution in the northern hemisphere in diverse environments such as private gardens, city centers, municipal parks, nature reserves, and forests.

To enhance our understanding about the diversity and function of microbial communities living in the phylloplane, culture-independent approaches are indispensable. Nevertheless, one of the key challenges for microbiologists remains to develop strategies to culture the vast diversity of microorganisms. There has been a recent resurgence in the application of classical culture techniques to interrogate the microbial world, with particular success in environments such as the human gut [12–14]. In general, a wide diversity of cultured bacteria may be retrieved by increasing the diversity of growth media used to include complex media rich in macro- and micronutrients, and custom media formulations that are more oligotrophic. This includes growth media with low concentrations of mineral salts [15, 16], the addition of (host) plant extracts [17], separated preparation of growth medium components [18], and the use of a range of solidifying agents [19]. Monitoring for colony formation over extended incubation periods is also useful [16]. Once a collection of bacterial isolates is obtained and maintained in the laboratory their functional characteristics can be evaluated, including plant growth-promoting (PGP) potential through the biosynthesis of PGP hormones and production of specific enzymes.

Here, we deployed microbial community metabarcoding to evaluate the culturing efficiency of bacteria from the *H. helix* phylloplane, specifically evaluating the use of Luria–Bertani (LB) [20], LB01 (1/10 dilution of LB), yeast extract–mannitol (YMA) [21], yeast extract–flour (YFlour) [22], and YEx (custom formulation). Additionally, for each growth medium, representative bacterial isolates were purified such that their PGP potential could be evaluated. This study highlights the usefulness of high-throughput sequencing to evaluate the diversity of bacterial communities present on growth media in comparison to uncultured samples from the original environment. The information obtained can guide targeted single-colony isolation, focusing on growth conditions that favour certain taxa thereby increasing the likeliness to isolate previously uncultured or underrepresented bacterial species.

## Results and discussion

### The *Hedera helix* phylloplane and its culturable fraction

**Fig. 1:**
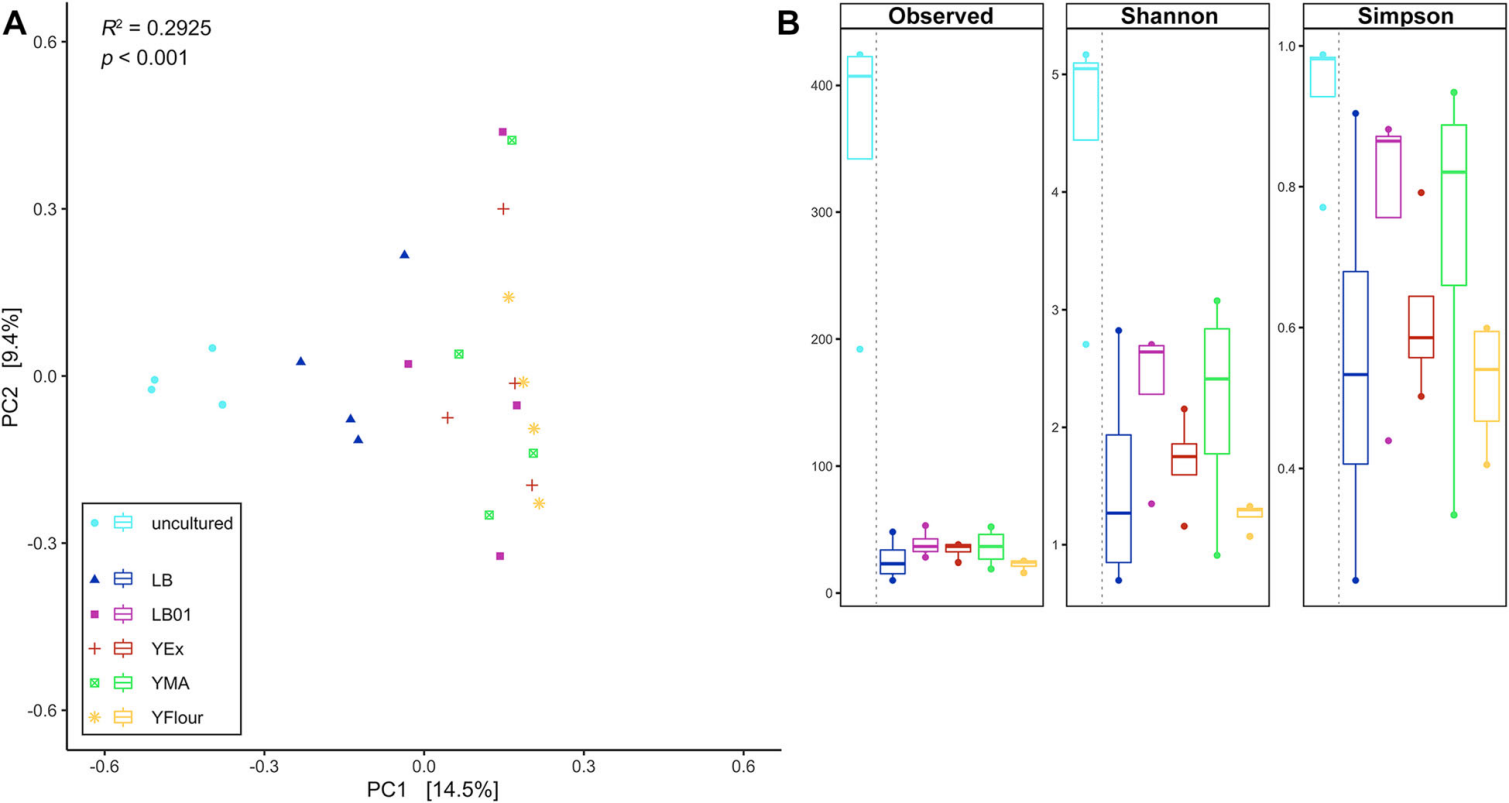
Intra- and inter-diversity of growth medium and phylloplane samples. Intra-sample diversity was assessed with amplicon sequence variant (ASV) observations, Shannon diversity and Simpson diversity (**b**; *n* = 24, 4 per growth medium and 4 uncultured phylloplane samples). Inter-sample diversity was measured with principal coordinates analysis (PCoA) on a Bray–Curtis dissimilarity matrix (**a**). The x- and y-axes are indicated by the first and second principal coordinate (PC), respectively, and the values in parentheses show the percentages of the variation explained

In an effort to expand the library of phylloplane bacterial isolates from *H. helix* host plants, phylloplane samples were analysed in a culture-dependent and -independent way. Metabarcoding was applied to picture the *H. helix* phylloplane bacterial diversity. Broad characterization of the culturable fraction, using both metabarcoding of Petri dish rinsates and Sanger sequencing of individual isolates, was carried out using five different growth media. The metabarcoding effort of both uncultured and cultured samples yielded 177,872 high-quality 300 bp V3–V4 16S rRNA gene sequences, representing 1482 amplicon sequence variants (ASVs). Bacterial intra-sample (alpha) diversity was estimated by rarefaction analysis (Figure A1) and by calculating three alpha diversity indices: (i) the observed number of ASVs, (ii) Shannon’s diversity index, and (iii) Simpson’s diversity index (Fig. 1b). The uncultured phylloplane samples showed the highest intra-sample diversity, while community diversity of cultures grown on LB01 and YMA was higher compared to LB, YEx, and YFlour. Overall, community diversity for all growth medium samples was low relative to uncultured phylloplane samples, as expected. To infer bacterial inter-sample (beta) diversity, we employed PCoA on a Bray–Curtis dissimilarity matrix (Fig. 1a). Statistical analysis revealed that the choice of growth medium contributes significantly to diversity (*R*^2^ = 0.2925, *p* < 0.001). Visual examination of the PCoA plot shows that inter-sample bacterial diversity shifts from LB, and to a lesser extent LB01, which contain the highest amount of resources (especially nitrogen sources), to YMA and YFlour, which are more selective, and to YEx, which is more limited but also most varied in resources. Differences in carbon-to-nitrogen ratio and carbon sources between the growth media likely contributed to the alpha and beta diversity and prevailing taxonomic groups observed, as it is the case in other bacterial (culture) systems [23–25].

**Fig. 2:**
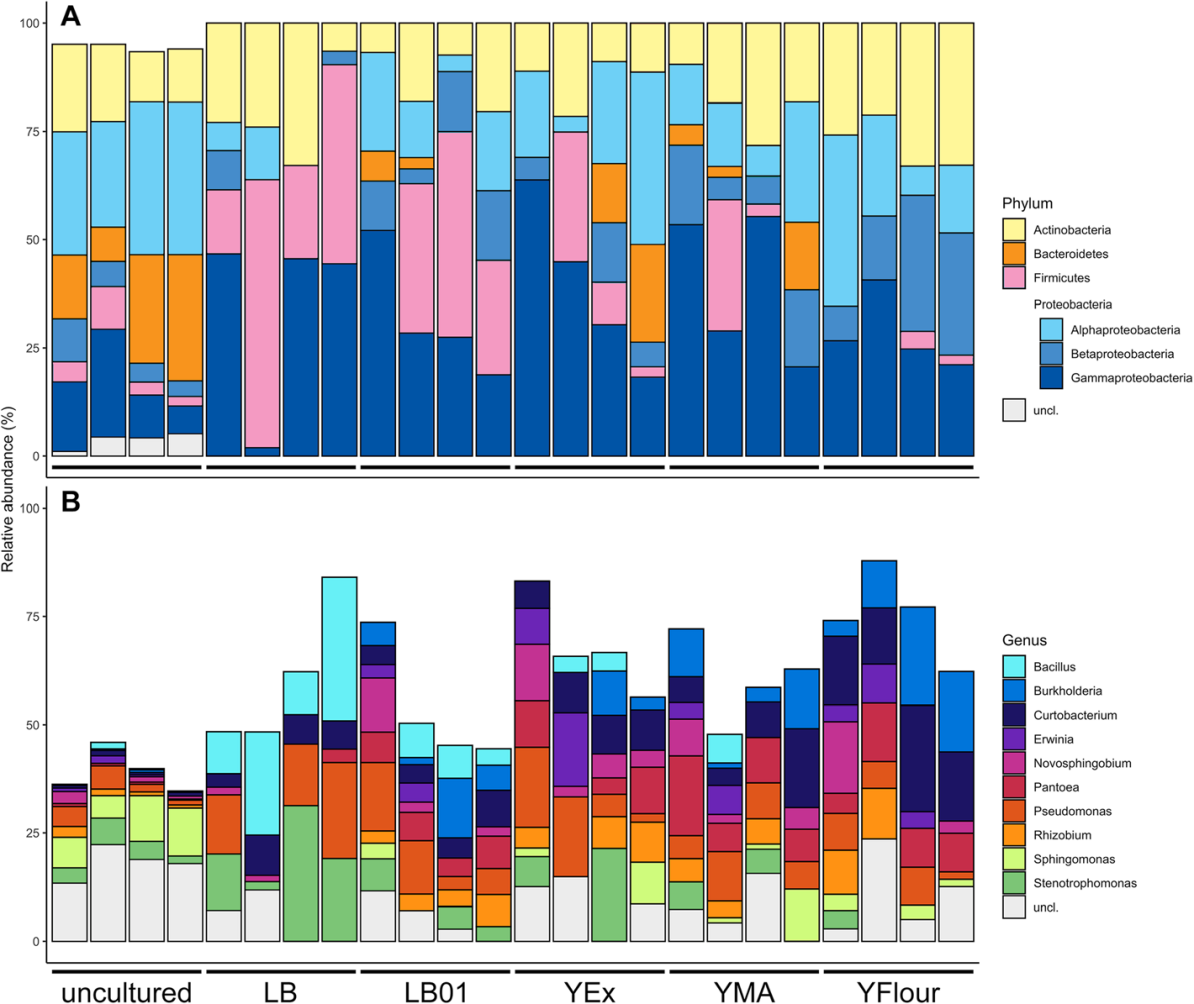
Taxonomic diversity of cultured phylloplane bacteria on the selected growth media and relation with the uncultured phylloplane. Relative abundances of the four major phyla Proteobacteria, Actinobacteria, Bacteroidetes and Firmicutes (**a**) across all samples (*n* = 24, 4 per growth medium and 4 uncultured phylloplane samples). The relative abundances of the top 10 genera across all growth medium samples and their relation with the uncultured bacterial phylloplane is also shown (**b**). uncl.: unclassified

In order to take a closer look at the composition of the bacterial communities from uncultured and cultured samples, ASVs were taxonomically assigned using the latest version of the Ribosomal Database Project (RDP) database. From this, differences between the uncultured phylloplane and the cultured samples were evident (Fig. 2). For uncultured phylloplane samples at the phylum level (Fig. 2a), an average of 90.7% of ASVs could be taxonomically classified within four major phyla with the following relative abundances: Proteobacteria (51.1%; subdivided as 30.9% Alphaproteobacteria, 14.9% Gammaproteobacteria, and 5.9% Betaproteobacteria), Actinobacteria (15.5%), Bacteroidetes (19.2%), and Firmicutes (4.9%). For the remaining 5.6%, ASVs were classified within 12 other phyla (Acidobacteria, Armatimonadetes, Chlamydiae, Cyanobacteria, Deinococcus–Thermus, Fusobacteria, Gemmatimonadetes, Nitrospirae, Planctomycetes, Saccharibacteria, Verrucomicrobia, and candidate phylum WPS-1). Finally, 3.7% could not be classified at phylum level. It was previously reported for different plant species, including *Arabidopsis thaliana* (thale cress), *Lactuca sativa* (lettuce), *Glycine max* (soy bean), *Trifolium repens* (white clover), and *Oryza sativa* (rice) that their phyllosphere communities are mainly comprised of bacteria belonging to phylum Proteobacteria (with classes Alphaproteobacteria and Gammaproteobacteria in particular), Actinobacteria, Bacteroidetes, and Firmicutes [1, 3–8]. Members of Proteobacteria constituted about 50% of the community composition. Here we showed that this holds true for *H. helix*. This strengthens the observation that the phyllosphere microbiome composition on higher taxonomic level is stabilized by factors such as host plant species and geographical location [1]. Host plant species can be the primary factor driving the composition of the phyllosphere microbiome [26, 27], while in other cases geographic location can have the greatest influence on community composition [4, 28, 29].

For growth medium samples, all ASVs were taxonomically classified within the phyla Proteobacteria, Actinobacteria, Bacteroidetes, and Firmicutes. This may be expected, given the overall taxonomic structure of the *H. helix* phylloplane and the general finding that the vast majority of cultured bacteria are affiliated with these phyla [30]. All selected growth media favoured Actinobacteria and Gammaproteobacteria, with average relative abundances of 19.0 and 34.7%, respectively, while Bacteroidetes were only recovered from LB01, YEx, and YMA. LB and LB01 favoured Firmicutes compared to the other growth media, with an average relative abundance of 36.1 and 27.1%, respectively. YFlour was most selective for Betaproteobacteria (20.6%). Figure 2b illustrates the relative abundances of the 10 most abundant genera across all growth media and the uncultured bacterial phylloplane. LB favoured the growth of *Bacillus* and *Stenotrophomonas*, with average relative abundances of 19.2 and 16.3%, respectively, while YFlour was most selective for *Burkholderia* (13.9%) and *Curtobacterium* (14.7%). *Rhizobium* was found on all growth media except on LB. In the uncultured phylloplane an average of 18.1% of ASVs could not be classified at the genus level. For growth medium samples this was 4.7% for LB, 5.4% for LB01, 9.0% for YEx, 11.0% for YFlour, and 6.8% for YMA. This suggests that more potentially novel bacterial species were cultured on YFlour, while LB and LB01 yielded the highest abundance of known bacteria.

**Fig. 3:**
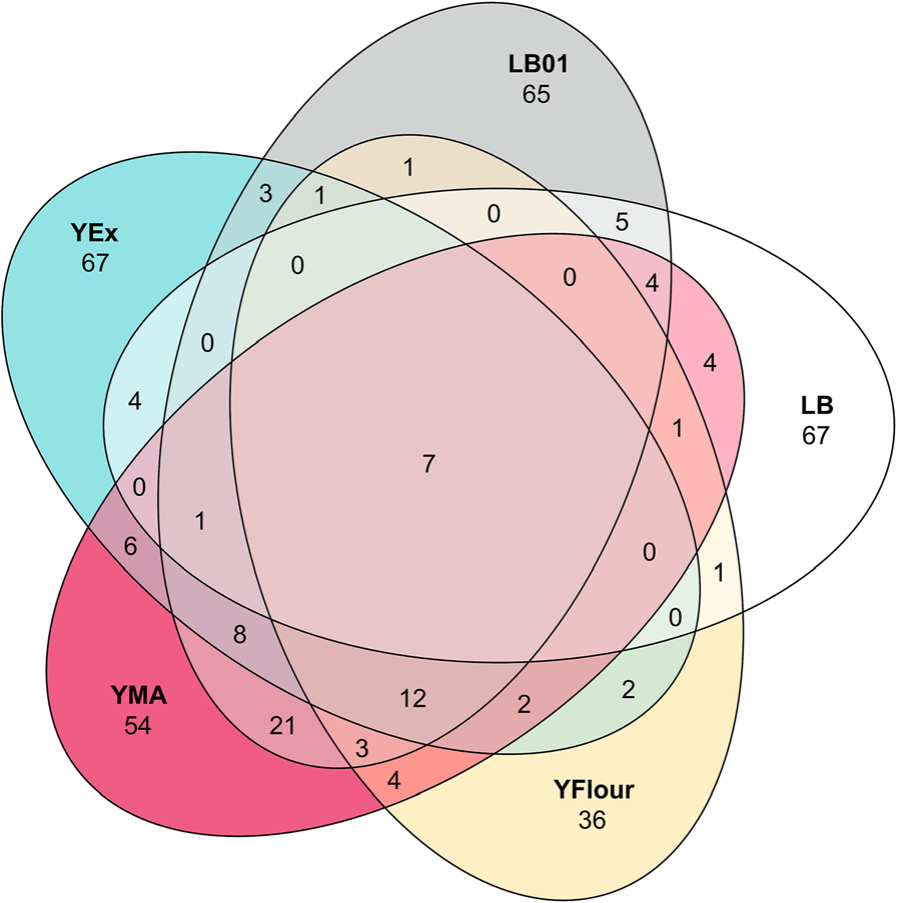
Venn diagram showing shared and unique amplicons sequence variants (ASVs) for the selected growth media. All 379 ASVs that were obtained by culturing are depicted according the selected growth media. The highest proportion of ASVs (76.3%) is unique for the growth media, 21.9% is shared between at least two of the growth media and only 1.8% is shared between all growth media. Note that ASV abundance is not considered in this diagram

**Fig. 4:**
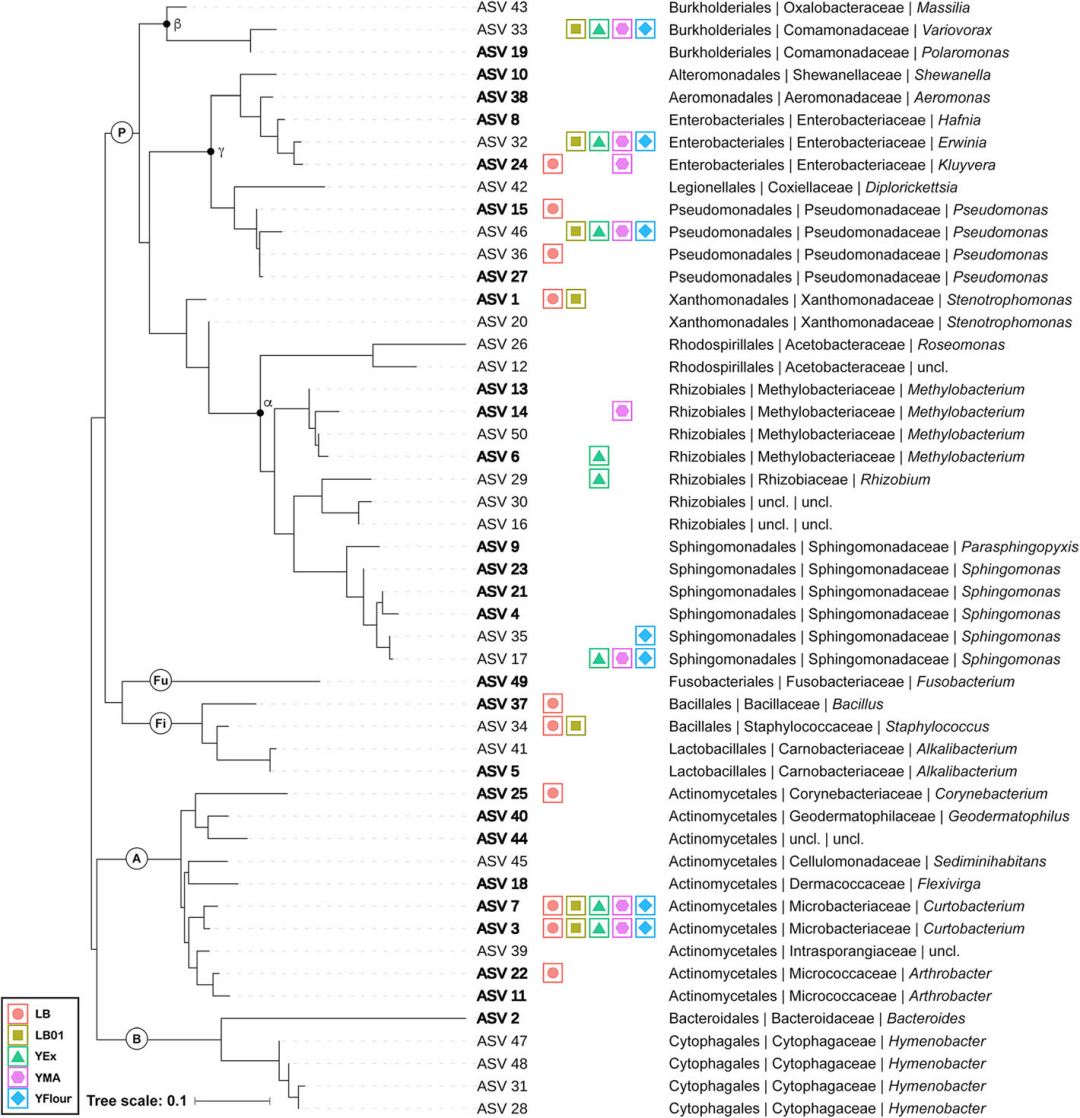
Most abundant amplicons sequence variants (ASVs) and relation with the selected growth media. The top 50 ASVs in uncultured bacterial phylloplane samples are given with their phylogenetic relationship and taxonomic classification (order | family | genus). ASVs depicted in bold are part of the core microbiome, here defined by presence in all phylloplane samples. For each ASV, it is indicated if culturing was successful on the selected growth media with a corresponding symbol. P: Proteobacteria, α: Alphaproteobacteria, β: Betaproteobacteria, γ: Gammaproteobacteria, A: Actinobacteria, B: Bacteroidetes, Fi: Firmicutes, Fu: Fusobacteria, uncl.: unclassified

To understand the importance of culturing conditions to capture a substantial part of the total phyllospheric bacterial community of *H. helix*, the proportion of shared and unique ASVs for each growth medium was determined. The highest proportion of ASVs (76.3%) was unique for the growth media, 21.9% was shared between at least two of the growth media and only 1.8% was shared between all growth media (Fig. 3). This exemplifies the added value of using varied growth conditions, such as different types of growth media, in the context of culturing a higher proportion of the microorganisms from a given environment. However, it is important to note that ASV abundance is not considered in this picture. The 50 most abundant ASVs in the uncultured bacterial phylloplane samples and their phylogenetic relationships are shown in Fig. 4. The growth conditions that were used allowed for culturing of 18 of the top 50 ASVs observed in uncultured samples. These ASVs all classified within the phyla Proteobacteria, Actinobacteria, Bacteroidetes, or Firmicutes except one (ASV 49) that was classified as *Fusobacterium* within the phylum Fusobacteria. Many of these ASVs classified as genera typically associated with the phyllosphere [1, 3–8], including *Bacteroides*, *Curtobacterium*, *Methylobacterium*, *Pseudomonas*, *Rhizobium*, *Sphingomonas*, and *Stenotrophomonas*.

**Fig. 5:**
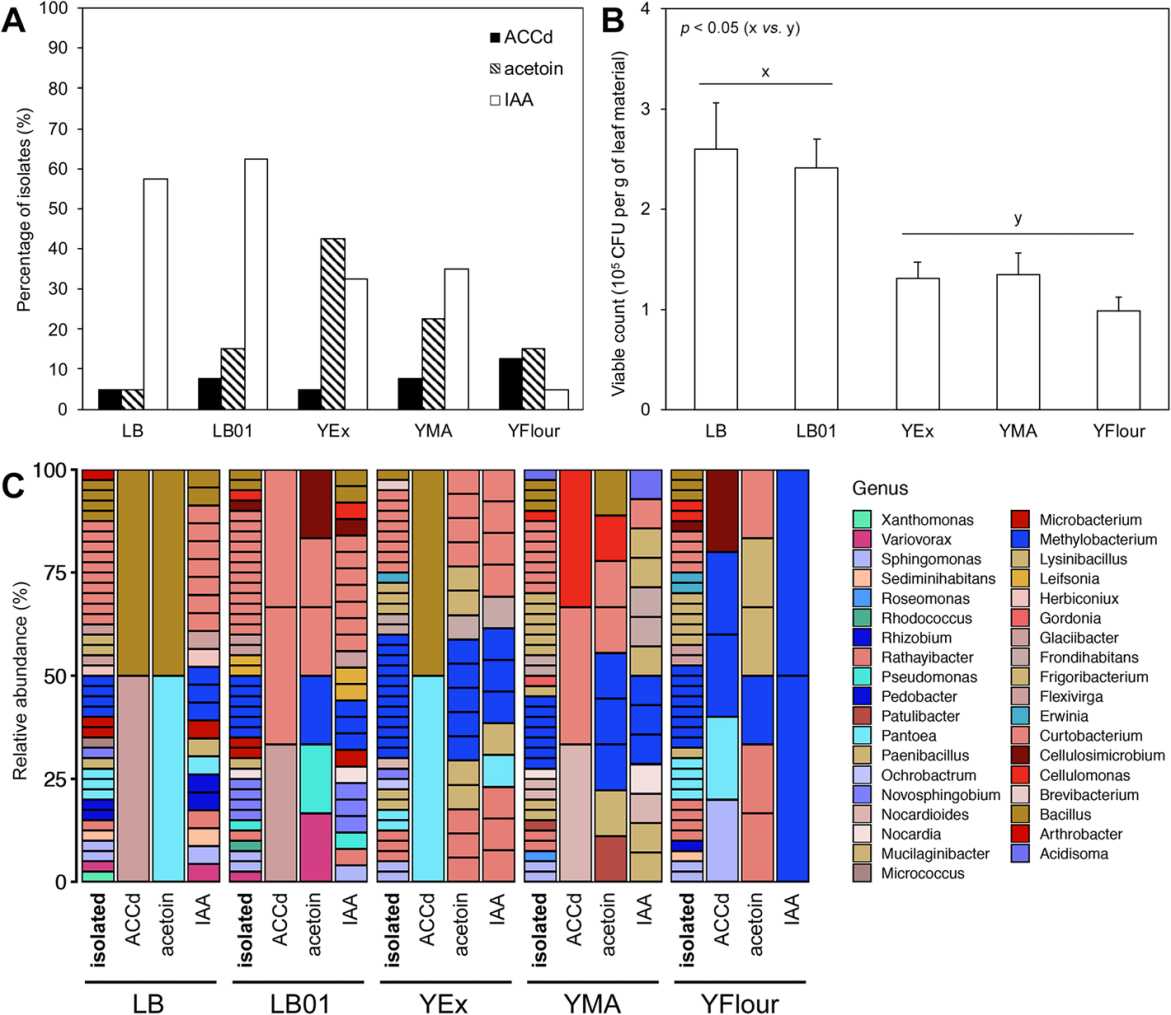
Plant growth-promoting potential and viable count of the selected growth media. Four weeks after inoculation, the number of colony-forming units (CFU) per gram of fresh leaf material on the growth media was determined (**b**; *n* = 40, 8 per growth medium), with “x” and “y” indicating two significantly different groups (*p* < 0.05). Plant growth-promoting (PGP) potential was evaluated for isolates from each growth medium (**a**; *n* = 200, 40 per growth medium). The taxonomic classification at the genus level of these 200 isolates tested for PGP potential is also shown (**c**). ACCd: 1-aminocyclopropane-1-carboxylic acid deaminase, acetoin: 3-hydroxy-2-butanone, IAA: indole-3-acetic acid

Another, more traditional, method to assess culturing efficiency is to count the viable bacterial colonies growing on Petri dishes. A comparison of the viable count of phylloplane colonies growing on the selected growth media, expressed in colony-forming units (CFU) per gram of fresh leaf material, is shown in Fig. 5b. LB and LB01 allowed for growth of significantly higher numbers of bacterial colonies compared to YEx, YFlour and YMA (*p* < 0.05). Not surprisingly, LB and LB01 contained the highest concentration of (nitrogen) resources, making it easier for *r*-selected species to grow.

### Plant growth-promoting potential of 200 bacterial isolates

Evaluation of PGP potential was determined as indole-3-acetic acid (IAA), 3-hydroxy-2-butanone (acetoin), and 1-aminocyclopropane-1-carboxylic acid (ACC) deaminase production by 200 bacterial isolates cultured with the selected growth media (Fig. 5a). IAA is the most common phytohormone of the auxin class, and induces cell elongation and division for plant growth and development [31]. The volatile phytohormone acetoin has been shown to promote growth and induce systemic resistance in *A. thaliana* [32, 33], and ACC deaminase reduces ethylene levels, which is related to plant growth promotion [34]. In our study regarding phylloplane bacteria, those capable of IAA production were abundant on LB and LB01, and nearly absent on YFlour. YEx yielded bacterial isolates showing relatively high production of acetoin. Isolates producing ACC deaminase were rare on all growth media. The bacterial 16S rRNA gene of all 200 isolates was partially sequenced and these sequences were taxonomically assigned to genus level (Fig. 5c). Most isolates were assigned to the genera *Curtobacterium* (41) and *Methylobacterium* (37). *Frigoribacterium* (16), *Bacillu*s (13), *Rathayibacter* (11), *Sphingomonas* (10), and *Pantoea* (9) were also common. That one-fifth of cultured bacteria were classified within the genus *Curtobacterium* may not be surprising, as this genus is ubiquitously reported to be associated within phyllosphere habitats [35–38]. In one comprehensive isolation study comprising 200 leaf samples of soybean and corn plants, *Curtobacterium* species could be isolated from every sample [39]. Also, previous culture-independent phyllosphere studies paired with isolation have allowed the identification of representative bacteria from various genera, including *Methylobacterium* [40], *Frigoribacterium* [36]*, Sphingomonas* [41, 42], and *Pantoea* [43]. Most isolated *Curtobacterium* and *Methylobacterium* species in this study were able to produce IAA and acetoin, while correlations with ACC deaminase production were not evident. PGP profiles can help to select bacterial isolates with specific PGP traits that can be exploited in microbe-assisted plant biomass production, plant protection, and phytoremediation [10]. However, it is important to note that evaluating PGP traits based on in vitro experiments solely has its caveats [44]. For example, it is possible that the production of phytohormones does not occur in the natural plant–microbe partnership or that production occurs in a pathogenic context [45]. Follow-up in vivo inoculation experiments are necessary to conclusively evaluate PGP potential, but nevertheless in vitro PGP screening remains an important first step.

### Expanding on current culture databases

Here we highlight the potential of simple strategies to obtain higher microbial diversity from environmental samples. Next to relatively minor tweaks such as the addition of (host) plant extracts [17] or the use of a range of incubation periods [16], we showed that the use of different growth media proves to be effective in order to capture a substantial part of the total phyllospheric bacterial community of *H. helix*. Recently, this strategy also proved to be successful in the human gut [14] and a dryland environment [46], and likely is applicable on a wider basis.

Our isolation of phylloplane bacteria resulted in a collection of 200 bacterial isolates underrepresented in current databases. Most isolates (104/200) were taxonomically classified within the phylum Actinobacteria, which represented 20 out of a total of 37 genera, including *Curtobacterium* (41), *Frigoribacterium* (16), *Rathayibacter* (11), *Glaciibacter* (5), *Cellulomonas* (4), *Frondihabitans* (4), *Microbacterium* (4), *Nocardioides* (3), *Cellulosimicrobium* (2), *Leifsonia* (2), *Nocardia* (2), *Sediminihabitans* (2), *Arthrobacter* (1), *Brevibacterium* (1), *Flexivirga* (1), *Gordonia* (1), *Herbiconiux* (1), *Micrococcus* (1), *Patulibacter* (1), and *Rhodococcus* (1). This is interesting given the fact that Actinobacteria members are well-known for their secondary metabolite production [47] and abundant occurrence in extreme environments, characterized by acidic/alkaline pH, low or high temperatures, salinity and radiation, and low levels of moisture and resources [48]. For example, *Frigoribacterium* and *Glaciibacter* are typical psychrophilic genera containing a rare group of B-type peptidoglycan [49, 50], and *Frondihabitans* species are well-adapted to colder and ultraviolet light-exposed environments such as the phylloplane [51].

Additionally, in the context of the coordinated efforts to expand our understanding about plant-associated bacteria and life in general, several isolates from this study were selected for whole-genome sequencing in the framework of the U.S. Department of Energy (DOE) Joint Genome Institute (JGI) project the “Genomic Encyclopedia of Bacteria and Archaea (GEBA)” [52]. A further (pan)genomic study including a comparison with all publicly available genomes to understand which properties are specific to the phylloplane is ongoing.

## Conclusions

This study gives first insights into the total bacterial community of the *H. helix* phylloplane and contributes as case study of bacterial culturability of this habitat assessed using high-throughput sequencing technology, including an evaluation of the growth media LB, LB01, YMA, YFlour, and YEx. We provide a collection of 200 bacterial isolates underrepresented in current databases, including the characterization of PGP profiles to facilitate better understanding of the putative ecological roles of phylloplane bacteria that can also guide selection of inoculants for plant–microbe biotechnologies. In the context of international microbial culture collection initiatives aiming to culture at least one member of each functionally diverse group of the microbiota associated with plant hosts, this study highlights the potential of simple strategies to obtain higher microbial diversity from environmental samples and the use of high-throughput sequencing to guide isolate selection from a variety of growth media.

## Methods

### Collection and preparation of phylloplane samples

Leaves (*n* = 80, 20 per site) from *H. helix* plants ranging in age from three to 6 months old were collected at four sites around Hasselt, Belgium (coordinates in WGS84 format: 50.936546, 5.317226 (A); 50.928680, 5.332674 (B); 50.940104, 5.438675 (C); 50.921694, 5.433951 (D)). The distances in km between the sites are: A–B, 1.4; A–C, 8.5; A–D, 8.4; B–C, 7.6; B–D, 7.2; C–D, 2.1. The soil type at the four sites was characterized to be sandy loam with an average pH of 6.4 ± 0.1 (A: 6.41 ± 0.02, B: 6.56 ± 0.02, C: 6.42 ± 0.01, D: 6.37 ± 0.02) and average soil organic matter content of 958 ± 154 mg kg^− 1^ (A: 766 ± 61 mg kg^− 1^, B: 932 ± 28 mg kg^− 1^, C: 1169 ± 35 mg kg^− 1^, D: 965 ± 26 mg kg^− 1^). Permission for sampling was obtained and performed in accordance with institutional and international guidelines. Plant leaves were identified as specimens belonging to *H. helix* by the first author, and verified by all co-authors; voucher specimens are available from Hasselt University. Leaves were cut from the plants at shoulder height using sterile forceps, put in sterile tubes (five leaves per tube) filled with autoclaved phosphate buffer (50 mM Na_2_HPO_4_∙7H_2_O, 50 mM NaH_2_PO_4_∙H_2_O, 0.8 mM Tween 80, pH 7.0) and immediately transferred to the laboratory. Leaf weight was determined gravimetrically and microbial cells were detached from the leaf surface by sonication (100 W, 42 kHz, 3 min), followed by shaking on an orbital shaker (240 rpm, 30 min). Next, 16 resulting leaf wash suspensions (four per site, each suspension resulted from five leaves) were centrifuged (4000 rpm, 15 min) and the resuspended pellets were randomly pooled into four samples. For each sample, an aliquot was immediately stored at − 80 °C until DNA isolation; another aliquot was stored overnight at 4 °C for culturing of phylloplane bacteria.

### Metabarcoding of the bacterial phylloplane

Leaf wash suspensions (*n* = 4) were centrifuged (13200 rpm, 20 min, 4 °C) and genomic DNA was isolated using a NucleoSpin Soil kit (Macherey–Nagel, Düren, Germany). The V3–V4 hypervariable region of the bacterial 16S rRNA gene was PCR-amplified using 341F (5′-CCTACGGGNGGCWGCAG-3′) and 785R (5′-GACTACHVGGGTATCTAATCC-3′) primers with attached GS FLX Titanium adaptors, the sequencing key TCAG, and a sample-specific multiplex identifier. PCR products were purified by gel electrophoresis (1.5% agarose gel, 90 V, 45 min) and the 514 bp bacterial amplicon was excised and further purified using the UltraClean GelSpin DNA extraction kit (Mo Bio Laboratories, Carlsbad, CA, USA). Samples were brought to an equimolar concentration (10^10^ molecules μL^− 1^) using the Quant-iT PicoGreen dsDNA assay kit (Thermo Fisher Scientific, Waltham, MA, USA). Correct amplicon size and integrity were checked on an Agilent 2100 Bioanalyzer system (Agilent Technologies, Santa Clara, CA, USA), followed by sequencing on a Genome Sequencer FLX system (Roche Applied Science, Penzberg, Germany) with GS FLX Titanium series reagents by Macrogen Europe (Amsterdam, The Netherlands).

### Characterization of cultured phylloplane bacteria

Leaf wash suspension aliquots of 10 μL (*n* = 4) were pooled, diluted 1/100 and inoculated on 120 × 120 mm square Petri dishes containing LB [20], LB01 (1/10 dilution of LB), YMA [21], YFlour [22], or YEx (this study) and incubated at 30 °C (10 replicates per growth medium). Gellan gum was used as solidifying agent because of its thermal stability and resistance to desiccation, which makes it possible to incubate at 30 °C for a longer time compared to agar [53]. Phosphate-containing components were autoclaved separately to prevent the formation of growth-inhibiting molecules such as H_2_O_2_ [18]. The compositions of the growth media are summarized in Table 1. Four weeks after inoculation, the number of CFU per gram of fresh leaf material was determined for eight replicates per growth medium. Subsequently, biomass was rinsed from the surface of each plate using sterile 10 mM MgSO_4_. Followed by centrifugation (4000 rpm, 15 min), resuspended pellets were pooled into four samples per growth medium. Pellets were immediately stored at − 80 °C until DNA isolation. Genomic DNA was isolated as described previously and the V3–V4 hypervariable regions of the bacterial 16S rRNA genes were PCR-amplified, purified and prepared for sequencing.

**Table 1:**
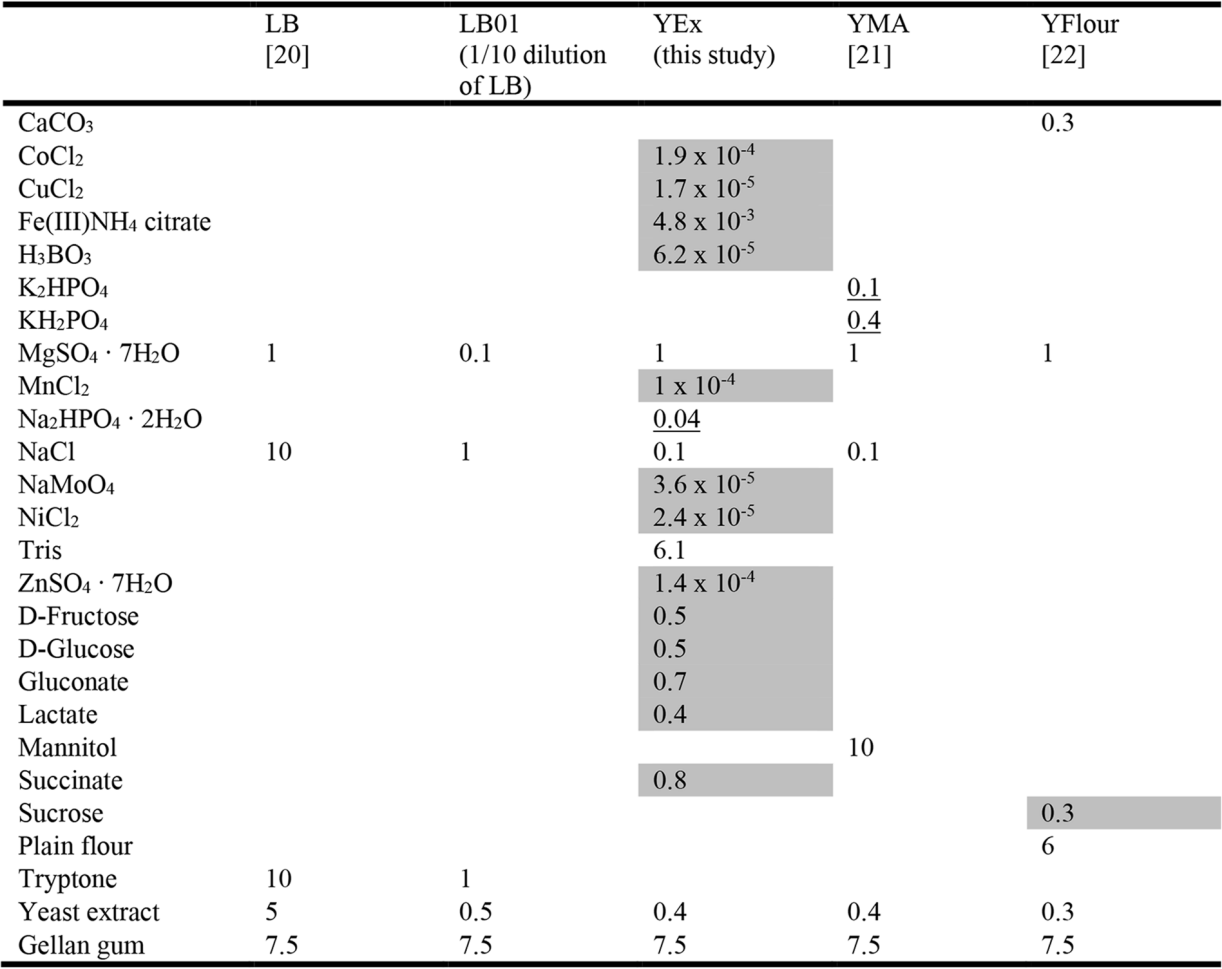
Composition of the growth media. Products are given in g L^− 1^ distilled water. Products marked in grey were filter-sterilized before being added to the other autoclaved components. Phosphate-containing components (underlined) were separately autoclaved. All growth media have pH 7.0

### Evaluation of plant growth-promoting potential

Bacterial phylloplane colonies were randomly picked from two replicates per growth medium. Isolated colonies (*n* = 200, 40 per growth medium) were checked for purity by streaking, and grown for 24 h in their respective liquid growth medium at 30 °C on a shaker (150 rpm), washed and resuspended in 2 mL of sterile 10 mM MgSO4 solution to obtain suspensions containing bacteria in mid-exponential phase (OD_600 nm_ = 0.4). Next, 20 μL of this bacterial suspension was used for the detection of IAA production using the Salkowski’s reagent method [54], for the detection of acetoin production using the Voges–Proskauer test [55], and for assessing ACC deaminase activity by monitoring the amount of α-ketobutyrate generated by the enzymatic hydrolysis of ACC [56]. Genomic DNA of all 200 isolates was extracted using a MagMAX DNA Multi-Sample Kit (Life Technologies, Carlsbad, CA, USA) and a MagMAX Express-96 Deep Well Magnetic Particle Processor (Life Technologies, Carlsbad, CA, USA). The portion of the bacterial 16S rRNA gene was PCR-amplified using 27F (5′-AGAGTTTGATCMTGGCTCAG-3′) and 1492R (5′-TACGGYTACCTTGTTACGACTT-3′) primers, and 20 μL of the PCR product was used for unidirectional Sanger sequencing using the 27F primer by Macrogen Europe (Amsterdam, The Netherlands).

### Processing of sequencing data

Sequencing data were received in FASTQ format with GS FLX Titanium adaptors and the sequencing key TCAG trimmed from all sequences, demultiplexed based on the sample-specific multiplex identifier and further processed with DADA2 1.12.1 [57] for single-end analysis. All reads were quality-filtered by truncation to 340 bp (discarding all reads with fewer than 340 bp), with subsequent trimming of 40 bp from the 5′-end with maxEE = 2 (maximum number of expected errors), resulting in a data set consisting only of high-quality V3–V4 16S rRNA gene sequences of exactly 300 bp with all irrelevant sequences removed. For error model learning, dereplication, sample inference and chimera removal, default parameter settings were used. Taxonomy was assigned to each resulting ASV with IDTAXA [58] using the RDP 16S rRNA training set v16 [59]. The ASV table with assigned taxonomy was imported into phyloseq 1.28.0 [60] for making phylogenetic bar charts, and for rarefaction and diversity analysis. Intra-sample diversity was assessed with ASV observations, Shannon diversity and Simpson diversity. Inter-sample diversity was measured with principal coordinates analysis (PCoA) on a Bray–Curtis dissimilarity matrix, and different outcomes were tested using permutational multivariate analysis of variance (PERMANOVA, 999 permutations). Phylogenetic tree construction was done with PhyML 3.1 [61] after alignment of the sequences with MUSCLE 3.8.31 [62]. Differences in CFU between growth media were tested with a Kruskal–Wallis test followed by pairwise comparisons using a Wilcoxon rank-sum test with Benjamini–Hochberg correction. MicEco 0.9.11 was used for making a Venn diagram. Sanger sequencing data were processed using sangerseqR 1.20.0 and sangeranalyseR 0.1.0 [63], and resulting high-quality sequences were taxonomically assigned with BLAST+ 2.9.0 [64] using the RDP 16S rRNA training set v16 [59]. All data handling was done within R 3.6.3 [65].

## Supplementary Information

**Additional file 1: Figure A1.** Rarefaction analysis of all samples. Rarefaction plots indicating the average number of amplicon sequence variants (ASVs) for uncultured bacterial phylloplane samples (A; *n* = 4) and growth medium samples (B; *n* = 20).

## Data Availability

The metagenomic libraries of the bacterial phylloplane communities and sequences of the cultured bacteria on all growth media are available from the Short Read Archive (SRA) of the National Center for Biotechnology Information (NCBI) under project accession number PRJNA626008 and individual FASTQ sample identifiers SAMN14614763–86. All partial 16S rRNA gene sequences obtained by Sanger sequencing are available from NCBI under accession numbers MT360054–253.

## Abbreviations

ACC: 1-aminocyclopropane-1-carboxylic acid
ASV: Amplicon sequence variant
CFU: Colony-forming unit
IAA: Indole-3-acetic acid
PGP: Plant growth promotion
