# Supplementary figures and images for: Diversity and plant growth-promoting potential of (un)culturable bacteria in the Hedera helix phylloplane
Source: BMC Microbiol. 2021 Feb 27;21:66. doi: 10.1186/s12866-021-02119-z (PMC7912551; doi:10.1186/s12866-021-02119-z)

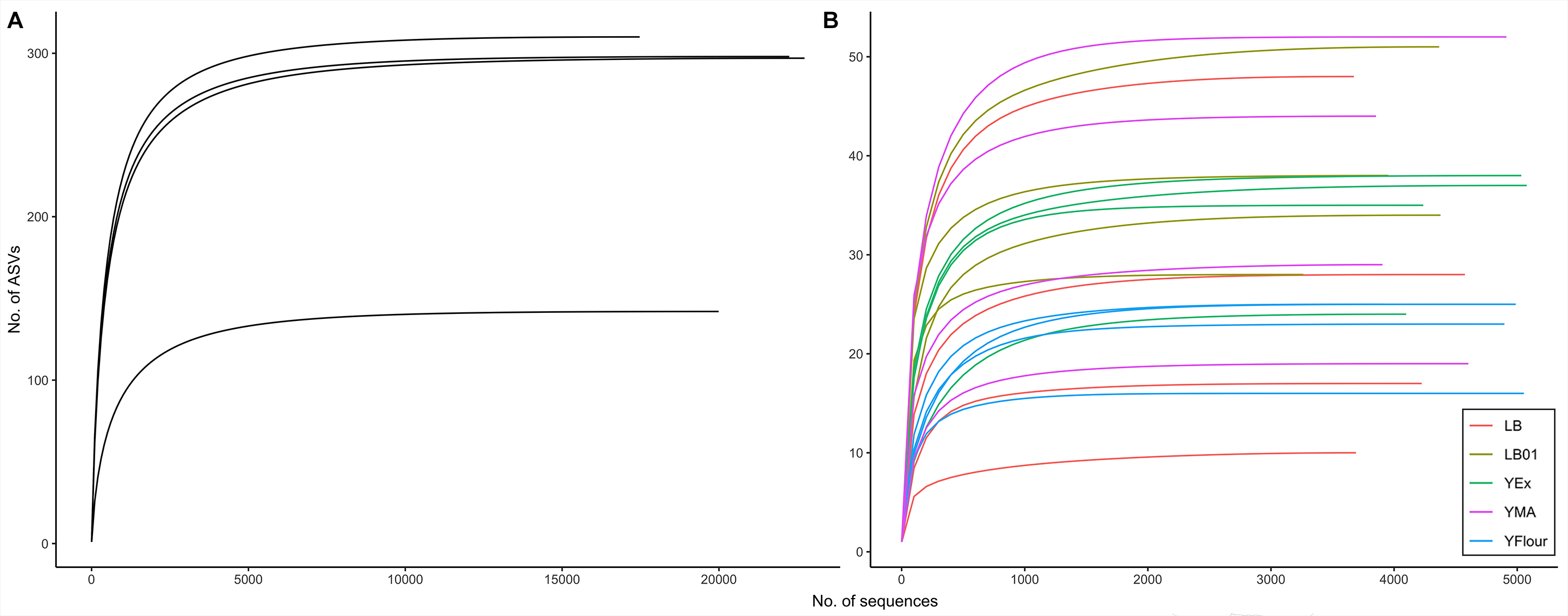

Supplement: Supplementary file 1 — Additional file 1: Figure A1. Rarefaction analysis of all samples. Rarefaction plots indicating the average number of amplicon sequence variants (ASVs) for uncultured bacterial phylloplane samples (A; n = 4) and growth medium samples (B; n = 20). [file 12866_2021_2119_MOESM1_ESM.tiff]
